# Scrub Typhus in the Modern Era: Clinical Spectrum, Organ Involvement, and Outcomes in a South Indian Tertiary Care Setting

**DOI:** 10.7759/cureus.110012

**Published:** 2026-05-31

**Authors:** Revathi K, Guranjan Kaur, Pruthvi J, Ranjith J

**Affiliations:** 1 Internal Medicine, Narayana Health City, Bengaluru, IND; 2 Internal Medicine, Narayana Hrudayalaya, Bengaluru, IND; 3 Internal Medicine, Sri Venkateshwaraa Medical College Hospital and Research Center, Tirupati, IND

**Keywords:** acute undifferentiated fever, ards, myocarditis, orientia tsutsugamushi, scrub typhus, south india

## Abstract

Background

Scrub typhus, an acute febrile illness caused by *Orientia tsutsugamushi*, is re-emerging in India, with varied clinical presentations and potentially life-threatening complications. However, data from southern India remain limited.

Aim

This study aimed to describe the clinical characteristics and outcomes of patients diagnosed with scrub typhus based on positive IgM antibody results detected by enzyme-linked immunosorbent assay (ELISA).

Methods

We conducted a prospective hospital-based observational study at a tertiary care center in Bengaluru from September 2022 to February 2024. Fifty adult patients with IgM ELISA-confirmed scrub typhus were enrolled. Demographic details, clinical features, laboratory parameters, complications, and outcomes were systematically recorded and analyzed.

Results

The mean age of patients was 50.5 ± 13.2 years, with equal male-to-female distribution (25 (50%) males and 25 (50%) females). Fever was present in 50 (100%) patients, followed by myalgia in 33 (66%) and gastrointestinal symptoms in 25 (50%) patients; eschar was detected in 12 (24%) patients. Laboratory abnormalities included elevated transaminases (AST > 3× upper limit of normal) in 15 (30%) patients, thrombocytopenia (<100 × 10³/μL) in 11 (22%) patients, and raised CRP (>5 mg/dL) in 48 (96%) patients. Major complications included acute respiratory distress syndrome (11, 22%), pneumonia (10, 20%), myocarditis (8, 16%), and acute kidney injury (6, 12%). Intensive care unit (ICU) admission was required in 39 (78%) patients, with ventilatory support required in 12 (24%) patients. All patients survived with appropriate treatment.

Conclusion

Scrub typhus presents with nonspecific clinical features and significant morbidity due to multi-organ involvement; however, favorable outcomes can be achieved with early recognition and timely treatment. Our findings emphasize the need for heightened clinical suspicion and routine testing for scrub typhus in patients with acute undifferentiated febrile illness in endemic regions.

## Introduction

Scrub typhus, also known as tsutsugamushi disease, is an acute febrile and potentially life-threatening zoonotic illness caused by *Orientia tsutsugamushi*, an obligate intracellular gram-negative bacterium. It is transmitted to humans through the bite of infected larval trombiculid mites, commonly known as chiggers, of the genus *Leptotrombidium* [1]. The disease is endemic to the “tsutsugamushi triangle,” an area extending from northern Japan and eastern Russia to northern Australia in the south, from India and Pakistan in the west, to the Pacific islands in the east [2]. Globally, over one billion people are at risk, with approximately one million cases reported annually [3].

In India, scrub typhus has emerged as a significant public health challenge, with cases reported across diverse ecological settings, ranging from Tamil Nadu and Karnataka in the south to Himachal Pradesh and Jammu and Kashmir in the north [4]. The clinical spectrum of the disease is broad, ranging from a mild self-limiting febrile illness to severe multi-organ dysfunction. Early symptoms are often nonspecific and include high-grade fever, headache, myalgia, and cough, frequently mimicking other tropical infections such as dengue, malaria, and leptospirosis [5]. A hallmark diagnostic sign is the eschar, a painless necrotic skin lesion at the site of the chigger bite; however, its prevalence varies significantly across different populations and skin types [6].

The pathophysiology involves widespread vasculitis resulting from infection of endothelial cells and subsequent perivascular infiltration. If left untreated, the inflammatory response can lead to severe complications, including acute respiratory distress syndrome (ARDS), acute kidney injury (AKI), myocarditis, meningoencephalitis, and multiple organ dysfunction syndrome (MODS), with mortality rates reaching up to 30% in untreated cases [7,8].

Despite its re-emergence and substantial disease burden, scrub typhus remains underrecognized because of a low index of suspicion among clinicians and the limited availability of confirmatory diagnostic tests in many settings. Early empirical therapy, primarily with doxycycline, is critical in reducing morbidity and mortality [9]. Furthermore, changing clinical presentations and the increasing use of advanced serological tests such as IgM enzyme-linked immunosorbent assay (ELISA) have highlighted the need for localized studies to better understand the current clinical spectrum and outcomes of the disease.

This study was conducted at a tertiary care hospital in Bangalore, Karnataka, to evaluate the contemporary clinical presentation, laboratory parameters, organ involvement, complications, and outcomes of patients diagnosed with scrub typhus in the era of improved serological diagnostics and advanced critical care management. By analyzing these factors, we aim to provide insights that may aid in earlier diagnosis and improved management strategies for this neglected tropical disease.

## Materials and methods

Study design and setting

This hospital-based, prospective, observational, cross-sectional study was conducted in the Department of General Medicine at Mazumdar Shaw Medical Centre, Narayana Health City, Bengaluru, Karnataka, India.

Study duration

The study was carried out over a period of 18 months from September 2022 to February 2024 after obtaining approval from the Institutional Ethics Committee.

Study population

Adult patients aged ≥18 years admitted under the Department of General Medicine with confirmed scrub typhus infection, diagnosed by positive IgM antibodies against *O. tsutsugamushi* using ELISA, were included in the study. Consecutive eligible patients admitted during the study period were enrolled after obtaining informed consent.

Sample size and sample size calculation

The sample size was calculated based on the study by Devasagayam et al. reporting the burden of scrub typhus in hospital-based Indian studies [4]. Assuming an estimated prevalence of 15%, a 5% level of significance, and an allowable error of 5%, the minimum required sample size was calculated using the standard formula for a single population proportion:

 $n = Z&sup2; &times; p(1 &minus; p) / d&sup2;$

where n is the required sample size, Z corresponds to the standard normal deviate at 95% CI (1.96), p is the estimated prevalence, and d is the allowable error.

Based on the calculation, the minimum sample size required was 49 patients. During the study period, a total of 50 eligible patients fulfilling the inclusion criteria and providing informed consent were enrolled consecutively in the study.

Inclusion and exclusion criteria

Patients aged ≥18 years admitted with positive IgM ELISA for *O. tsutsugamushi* were included in the study. Patients who were unwilling to participate in the study were excluded.

Data collection

Demographic details, clinical history, comorbid conditions, and presenting symptoms were recorded using a structured data collection proforma. Hypertension, diabetes mellitus, chronic kidney disease, and other comorbidities were defined based on documented prior diagnosis or ongoing treatment history.

All patients underwent a detailed clinical examination at admission. Laboratory investigations included complete blood count, liver function tests, renal function tests, serum electrolytes, C-reactive protein levels, arterial blood gas analysis, urine routine microscopy, blood and urine cultures where indicated, chest radiography, electrocardiography, ultrasonography of the abdomen and pelvis, and two-dimensional echocardiography when clinically indicated. Echocardiography was performed in patients with clinical suspicion of cardiac involvement, including chest pain, hypotension, arrhythmias, elevated cardiac biomarkers, or unexplained dyspnea.

Scrub typhus diagnosis was established using IgM ELISA for *O. tsutsugamushi* according to the manufacturer’s instructions and institutional laboratory standards. The test was performed in patients presenting with acute febrile illness clinically suggestive of scrub typhus.

Treatment protocol

All patients received doxycycline as first-line therapy at a dose of 100 mg twice daily. The duration of treatment was individualized based on clinical response. Supportive management, including intravenous fluids, oxygen therapy, ventilatory support, vasopressors, renal replacement therapy, and blood product transfusion, was provided as clinically indicated. Co-infections were managed according to standard institutional treatment protocols.

Outcome measures

The primary outcome measures included clinical presentation, laboratory parameters, complications, requirement for intensive care support, and in-hospital outcomes among patients diagnosed with scrub typhus. Secondary outcome measures included seasonal variation in disease occurrence and the incidence of complications like myocarditis and acute respiratory distress syndrome (ARDS).

Definitions of complications

Complications were defined using standard diagnostic criteria, as summarized in Table 1.

**Table 1:** Standard diagnostic criteria used for defining complications in patients with scrub typhus

Complication	Definition used in the study
Shock	Systolic blood pressure < 90 mmHg for at least one hour despite adequate fluid resuscitation
Acute kidney injury (AKI)	Defined according to the Kidney Disease: Improving Global Outcomes (KDIGO) criteria as an increase in serum creatinine ≥ 0.3 mg/dL within 48 hours, or ≥50% increase from baseline, or urine output < 0.5 mL/kg/hr for more than 6 hours
Acute respiratory distress syndrome (ARDS)	Defined according to the Berlin criteria as an acute onset within 7 days of clinical insult, bilateral chest infiltrates, and PaO₂/FiO₂ ≤ 300, with PEEP or CPAP ≥ 5 cm H₂O
Myocarditis	Elevated creatine kinase-MB above baseline and/or new-onset arrhythmia without prior history of atrial fibrillation, supraventricular tachycardia, or frequent premature ventricular contractions
Hepatitis	Elevation of AST or ALT more than five times the upper limit of normal or total bilirubin > 2 mg/dL
Central nervous system involvement (meningoencephalitis)	Altered sensorium with signs of meningeal irritation and cerebrospinal fluid showing elevated protein with lymphocytic/neutrophilic cytology and normal or low glucose
Disseminated intravascular coagulation (DIC)	Prolonged PT and/or aPTT, platelet count < 100 × 10⁹/L or rapid decline in platelet count within 24 hours, presence of schistocytes, and elevated fibrin degradation products
Thrombocytopenia	Platelet count ≤ 20,000/µL
Pancreatitis	Acute abdominal pain with elevation of serum amylase or lipase > 3× the upper limit of normal and/or radiological evidence of pancreatitis
Pneumonia	Presence of parenchymal lung lesion on chest radiography

Statistical analysis

Data were entered into Microsoft Excel (Microsoft Corp., Redmond, WA, USA) and analyzed using IBM SPSS Statistics for Windows, Version 20.0 (Released 2011; IBM Corp., Armonk, NY, USA). Continuous variables were tested for normality using the Shapiro-Wilk test and expressed as mean ± standard deviation or median with interquartile range as appropriate. Categorical variables were expressed as frequencies and percentages. Associations between categorical variables were assessed using the chi-square test or Fisher’s exact test, wherever appropriate. Missing data, wherever present, were excluded from the final analysis.

Ethical considerations

Ethical approval for the study was obtained from the Institutional Ethics Committee of Mazumdar Shaw Medical Centre, Narayana Health City, Bengaluru. Written informed consent was obtained from all participants prior to enrolment.

## Results

A total of 50 adult patients diagnosed with scrub typhus, confirmed by positive IgM ELISA, were included in this prospective observational cross-sectional study conducted over a period of 18 months from September 2022 to February 2024.

Demographic characteristics**

The mean age of the study population was 50.52 ± 13.28 years (median: 51 years, IQR: 18.75, range: 21-76 years) (Table 2). The majority of patients belonged to the 41-60 years age group (26, 52%), followed by 61-70 years (11, 22%), 31-40 years (7, 14%), 21-30 years (4, 8%), and 71-76 years (2, 4%). The study population showed an equal gender distribution, with 25 (50%) males and 25 (50%) females, resulting in a male-to-female ratio of 1:1 (Table 2).

**Table 2:** Demographic and clinical profile of patients with scrub typhus Continuous variables are expressed as mean ± standard deviation (SD), median, interquartile range (IQR), and range. p-values shown correspond to the Shapiro-Wilk test for assessment of normality of distribution.

Parameters	n	Mean	SD	Median	IQR	Min	Max	p-value
Age (years)	50	50.52	13.28	51.00	18.75	21.00	76.00	0.595
Scrub IgM titer	50	1.75	0.75	1.65	1.16	0.52	3.50	0.140
Clinical presentation								
Duration of fever (days)	50	8.10	4.86	7.00	6.00	1.00	20.00	0.004
Systolic blood pressure (BP) (mmHg)	50	116.46	20.49	111.00	24.75	80.00	180.00	0.015
Diastolic BP (mmHg)	50	71.84	13.63	70.00	20.00	50.00	100.00	0.044
Pulse rate (beats/min)	50	99.82	15.58	99.50	24.25	66.00	132.00	0.504
Temperature (°F)	50	98.53	1.00	98.00	1.00	97.80	103.00	<0.001
Respiratory rate (cycles/min)	50	22.64	5.67	20.00	10.00	16.00	40.00	<0.001
Oxygen saturation (%)	50	91.80	7.63	95.00	10.00	70.00	100.00	<0.001
Glasgow Coma Scale score	50	14.98	0.14	15.00	0.00	14.00	15.00	<0.001
Lab parameters								
pH	50	7.25	1.05	7.40	0.08	7.35	7.56	<0.001
Total leukocyte count (/cumm)	50	8.89	4.07	8.55	4.33	2.10	24.80	0.001
Platelet count (/cumm)	50	137.50	77.41	150.00	72.75	1.5 lakh	4.5 lakh	0.008
C-reactive protein (mg/L)	50	106.70	90.94	77.18	126.35	1.80	315.00	<0.001
Serum creatinine (mg/dL)	50	1.00	0.80	0.78	0.49	0.43	4.60	<0.001
Blood urea nitrogen (mg/dL)	50	16.78	13.45	12.00	9.50	3.00	67.00	<0.001
Serum sodium (mEq/L)	50	133.48	6.77	134.00	6.50	106.00	146.00	<0.001
Serum potassium (mmol/L)	50	4.20	0.63	4.10	0.80	3.10	6.60	0.008
Serum calcium (mmol/L)	50	7.83	0.69	7.82	1.13	6.40	9.33	0.793
Total bilirubin (mg/dL)	50	1.67	1.81	1.00	1.31	0.30	9.03	<0.001
Direct bilirubin (mg/dL)	50	0.53	1.05	0.10	0.37	0.00	5.38	<0.001
Aspartate aminotransferase (AST) (U/L)	50	154.68	128.69	119.50	137.00	31.00	616.00	<0.001
Alanine aminotransferase (ALT) (U/L)	50	113.92	86.16	86.00	104.75	20.00	333.00	<0.001
Alkaline phosphatase (IU/L)	50	221.56	202.28	149.00	228.00	52.00	976.00	<0.001
Gamma-glutamyl transpeptidase (IU/L)	50	161.76	147.45	111.50	121.00	16.00	674.00	<0.001
Intensive care								
Non-invasive ventilation (NIV) duration (days)	12	2.25	1.91	2.00	1.00	1.00	8.00	<0.001
Mechanical ventilation duration (days)	3	2.00	-	2.00	-	0.00	2.00	-
Intensive care unit (ICU) stay (days)	27	3.81	1.81	4.00	2.00	1.00	8.00	0.007
Length of hospital stay (days)	50	3.56	1.96	4.00	3.00	1.00	10.00	0.024

Seasonal distribution**

**Figure 1:**
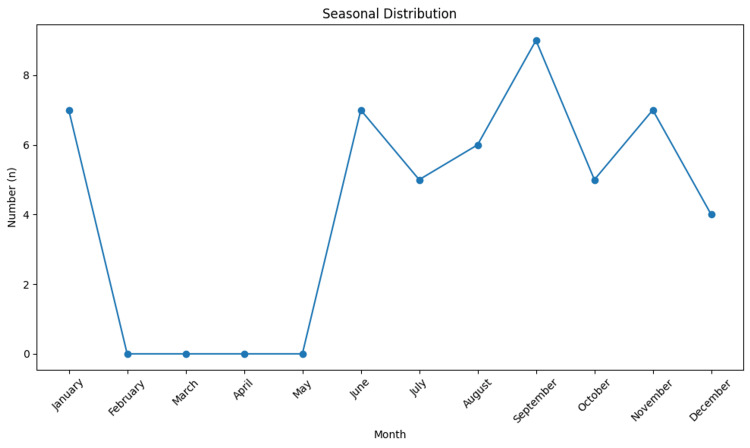
Distribution of patients according to the month of diagnosis The seasonal distribution of cases showed clustering during the monsoon and post-monsoon months, with the highest number of cases recorded in September (9, 18%).

Scrub typhus cases were predominantly observed during the monsoon and post-monsoon months, demonstrating a clear seasonal clustering pattern (Figure 1). The highest number of cases occurred in September (9, 18%), followed by July (7, 14%), November (7, 14%), and January (7, 14%). Additional cases were recorded in August (6, 12%), October (5, 10%), June (5, 10%), and December (4, 8%). Notably, no cases were reported between February and May during the study period.

Clinical presentation**

Fever was present in all patients (50, 100%), making it the most common presenting symptom. The mean duration of fever prior to presentation was 8.10 ± 4.86 days. Other presenting symptoms included myalgia in 33 (66%) patients, nausea and vomiting in 25 (50%), headache in 19 (38%), cough in 16 (32%), shortness of breath in 13 (26%), diarrhea in 11 (22%), pedal edema in 10 (20%), and abdominal pain in 9 (18%) patients. Less frequent symptoms included decreased urine output in three (6%) patients and skin rash, seizures, chest pain, and hematuria in one (2%) patient each. An eschar was identified in 12 (24%) patients (Table 3).

**Table 3:** Clinical presentation of patients with scrub typhus at admission

Clinical feature	Number of patients (n = 50)	Percentage (%)
Fever	50	100
Myalgia	33	66
Nausea and vomiting	25	50
Headache	19	38
Cough	16	32
Shortness of breath	13	26
Diarrhea	11	22
Pedal edema	10	20
Abdominal pain	9	18
Decreased urine output	3	6
Skin rash	1	2
Seizures	1	2
Chest pain	1	2
Hematuria	1	2
Eschar	12	24

Vital parameters at presentation**

At presentation, the mean systolic blood pressure and diastolic blood pressure of the study participants were 116.46 ± 20.49 mmHg and 71.84 ± 13.63 mmHg, respectively. The mean pulse rate was 99.82 ± 15.58 beats per minute, while the mean respiratory rate was 22.64 ± 5.67 cycles per minute. The mean body temperature recorded at admission was 98.53 ± 1.00°F, and the mean oxygen saturation was 91.80 ± 7.63%. The mean Glasgow Coma Scale (GCS) score at presentation was 14.98 ± 0.14, indicating preserved sensorium in most patients at admission (Table 2).

Laboratory parameters**

Laboratory evaluation of the study participants showed elevated inflammatory and biochemical markers at presentation (Table 2). The mean scrub typhus IgM ELISA titer was 1.75 ± 0.75. The mean C-reactive protein level was 106.70 ± 90.94 mg/dL. The mean serum creatinine and blood urea nitrogen levels were 1.00 ± 0.80 mg/dL and 16.78 ± 13.45 mg/dL, respectively. Electrolyte analysis revealed a mean serum sodium level of 133.48 ± 6.77 mEq/L, indicating hyponatremia as a common laboratory abnormality, while the mean serum potassium and serum calcium levels were 4.20 ± 0.63 mmol/L and 7.83 ± 0.69 mmol/L, respectively. Liver function tests demonstrated elevated transaminases, with mean aspartate transaminase (AST) and alanine transaminase (ALT) levels of 154.68 ± 128.69 U/L and 113.92 ± 86.16 U/L, respectively. The mean total bilirubin and direct bilirubin levels were 1.67 ± 1.81 mg/dL and 0.53 ± 1.05 mg/dL, respectively. Additionally, the mean alkaline phosphatase and gamma-glutamyl transpeptidase levels were 221.56 ± 202.28 IU/L and 161.76 ± 147.45 IU/L, respectively.

Cardiac evaluation findings

Electrocardiographic abnormalities were observed in several patients, with sinus tachycardia being the most common finding. Other abnormalities included conduction disturbances and arrhythmias in a smaller proportion of patients. Additionally, two-dimensional echocardiography revealed abnormalities suggestive of myocardial involvement consistent with myocarditis in a subset of patients, indicating cardiac involvement as one of the important complications associated with scrub typhus.

Complications

Complications were observed in 20 (40%) patients. The most common complications included ARDS in 11 (22%) patients, pneumonia in 10 (20%), myocarditis and shock in 8 (16%) patients each, and AKI in 6 (12%) patients. Central nervous system involvement and hepatitis were observed in 3 (6%) patients each, while disseminated intravascular coagulation (DIC) was noted in 2 (4%) patients (Table 4).

**Table 4:** Distribution of patients according to the complications

Complication	Number of patients (n = 50)	Percentage (%)
Acute respiratory distress syndrome (ARDS)	11	22
Pneumonia	10	20
Myocarditis	8	16
Shock	8	16
Acute kidney injury (AKI)	6	12
Central nervous system involvement	3	6
Hepatitis	3	6
Disseminated intravascular coagulation (DIC)	2	4

Co-infection

Co-infection with other infectious diseases was identified in nine (18%) patients. The most common co-infection was dengue IgM positivity in five (10%) patients, followed by urinary tract infection in two (4%) patients. Other co-infections included dengue NS1 antigen positivity in one (2%) patient, leptospirosis serology positivity in one (2%) patient, equivocal leptospirosis serology in one (2%) patient, COVID-19 infection in one (2%) patient, and H1N1 infection in one (2%) patient (Table 5).

**Table 5:** Distribution of co-infections among patients with scrub typhus

Co-infection	Number (n = 50)	Percentage (%)
Dengue NS1 Ag	1	2
Dengue IgM	5	10
Leptospirosis serology positive	1	2
Leptospirosis serology equivocal	1	2
Urinary tract infection	2	4
COVID-19	1	2
H1N1	1	2
Total	9	18%

Among them, three (33.3%) patients developed complications. However, there was no statistically significant association between co-infection and complications (chi-square test, p = 0.477) (Table 6).

**Table 6:** Association between co-infection and occurrence of complications in patients with scrub typhus Values are expressed as number (percentage). Association between categorical variables was assessed using the chi-square test. No statistically significant association was observed between co-infection and complications (p = 0.477).

Co-infection	Complications present, n (%)	Complications absent, n (%)	Total n (%)
Present	3 (33.3%)	6 (66.7%)	9 (18%)
Absent	17 (41.46%)	24 (58.54%)	41 (82%)
Total	20 (40%)	30 (60%)	50 (100%)

Treatment outcome

All patients received doxycycline as first-line therapy at a dose of 100 mg twice daily. Treatment was initiated promptly upon clinical suspicion/diagnosis and continued for a mean duration of seven days. Supportive and adjunctive therapies, including intravenous fluids, antipyretics, oxygen supplementation, ventilatory support, vasopressors, renal replacement therapy, and blood product transfusion, were administered as clinically indicated. Intensive care unit (ICU) admission was required in 39 (78%) patients (Table 2). Among these, mechanical ventilation was required in 3 (6%) patients, while non-invasive ventilatory support was required in 12 (24%) patients. Importantly, no mortality was observed in this study cohort, and all 50 (100%) patients were discharged following clinical recovery.

## Discussion

Scrub typhus is an important cause of acute undifferentiated febrile illness in India and continues to be associated with significant morbidity due to multi-organ involvement if diagnosis and treatment are delayed. The present prospective observational study evaluated the clinical spectrum, laboratory profile, complications, seasonal distribution, and outcomes among patients with scrub typhus admitted to a tertiary care hospital in Bengaluru, Karnataka. 

In the present study, the mean age of the patients was 50.52 ± 13.28 years, with the majority belonging to the 41-60 years age group (26, 52%), indicating that scrub typhus predominantly affected middle-aged individuals in this cohort. Similar findings were reported by Philomena et al. and Sharma et al., where most patients belonged to the adult and middle-aged population, indicating that scrub typhus commonly affects the economically productive age group in endemic regions [10,11]. 

The study demonstrated equal gender distribution (male-to-female ratio = 1:1), which is consistent with findings reported in several Indian hospital-based studies. However, some studies from rural settings have reported female predominance due to increased occupational exposure during agricultural activities [10]. 

Fever was present in all patients (50, 100%), making it the most consistent presenting symptom, followed by myalgia in 33 (66%) patients, nausea and vomiting in 25 (50%) patients, and headache in 19 (38%) patients. Similar clinical presentations have been reported in earlier studies by Varghese et al. from South India and Narvencar et al. from Goa [5,8]. These findings reaffirm that scrub typhus should be considered an important differential diagnosis in patients presenting with acute febrile illness in endemic areas. 

The presence of an eschar was noted in 12 (24%) patients in the present study. This observation is comparable with previous Indian studies reporting variable prevalence ranging from 12% to 45% [5,8]. The variability in eschar detection may be related to differences in skin pigmentation, anatomical location of lesions, and carefulness of clinical examination. This underlines the limitations of relying solely on eschar for diagnosis in Indian populations, particularly in rural settings where darker skin tones and atypical lesion sites may contribute to under-recognition. This highlights the importance of accessible and reliable serological tests, such as IgM ELISA, in endemic regions. 

Seasonal variation was evident in the present study, with most cases occurring during the monsoon and post-monsoon months, particularly in September (9, 18%), followed by July (7, 14%) and November (7, 14%). Similar seasonal clustering has been reported in several Indian studies and is likely related to increased vector activity during periods of higher rainfall and vegetation density, which facilitates transmission of scrub typhus in endemic regions [4,8]. 

Laboratory findings in the present study demonstrated elevated liver enzymes, thrombocytopenia, raised inflammatory markers, and hyponatremia, which are well-recognized laboratory abnormalities in scrub typhus. Comparable findings were reported by Varghese et al., who observed elevated transaminases in 87% and thrombocytopenia in 79% of patients [5]. These laboratory abnormalities serve as useful supportive indicators in suspected cases of scrub typhus. 

Complications were observed in 20 (40%) patients in the present study. The most common complications included acute respiratory distress syndrome in 11 (22%) patients, pneumonia in 10 (20%), myocarditis in 8 (16%), and shock in 8 (16%), followed by AKI in 6 (12%), central nervous system involvement in 3 (6%), hepatitis in 3 (6%), and disseminated intravascular coagulation in 2 (4%) patients. Similar complication profiles have been reported in previous Indian studies, where respiratory involvement and multi-organ dysfunction were among the most frequent severe manifestations of scrub typhus [7,12]. 

Cardiac involvement in the form of myocarditis was observed in 8 (16%) patients, while ARDS was noted in 11 (22%) patients in the present study, indicating significant cardiopulmonary involvement among hospitalized patients with scrub typhus. These findings are clinically important, as both myocardial involvement and severe respiratory complications are often under-recognized manifestations of scrub typhus and may contribute to adverse outcomes if not identified and managed early [13]. 

Co-infection with other infectious diseases was observed in 9 (18%) patients, with dengue infection being the most common co-infection. However, no statistically significant association was observed between co-infection and the occurrence of complications in the present study (chi-square test, p = 0.477). Although co-infections are known to potentially worsen clinical outcomes and delay recovery in patients presenting with acute febrile illness in endemic regions, the absence of a significant association in this study may be related to the relatively small sample size. Nevertheless, these findings highlight the importance of evaluating patients with acute febrile illness for concurrent infections in endemic settings to ensure timely diagnosis and appropriate management [4]. 

In the present study, 27 (54%) patients required ICU admission, of whom 12 (24%) required non-invasive ventilation and 3 (6%) required mechanical ventilation, indicating substantial disease severity among hospitalized patients with scrub typhus. Similar ICU admission rates have been reported in previous studies involving patients with complicated scrub typhus infection, highlighting the potential for significant organ involvement requiring advanced supportive care in severe cases [7,12]. 

Despite the occurrence of significant complications, most patients recovered with appropriate antibiotic therapy and supportive management. Early diagnosis and timely initiation of doxycycline remain crucial in preventing disease progression and reducing mortality [6]. 

Overall, the findings of the present study reinforce the importance of maintaining a high index of suspicion for scrub typhus in patients presenting with acute febrile illness, especially during monsoon seasons in endemic regions. Recognition of characteristic clinical features, laboratory abnormalities, and potential complications can facilitate early diagnosis and prompt treatment, thereby improving patient outcomes. 

From a public health perspective, the findings underscore the urgent need to integrate scrub typhus into routine febrile illness diagnostic algorithms in endemic regions. Misdiagnosis and delayed treatment continue to contribute to morbidity and mortality. Preventive strategies should include strengthening surveillance, improving laboratory capacity at peripheral centers, and raising awareness among primary healthcare providers. These measures are critical, as scrub typhus is one of the few treatable causes of acute febrile illness in South Asia [14] 

Strengths and limitations**

The strengths of this study include its prospective design, use of standardized definitions for complications, and focus on systemic and multi-organ involvement in scrub typhus. Limitations include the single-center setting and relatively small sample size, which may restrict generalizability. As this was a tertiary-care inpatient cohort, referral bias and overrepresentation of severe cases are possible. Post-discharge follow-up was not performed, limiting assessment of long-term sequelae.

Additionally, scrub typhus IgM ELISA may yield false-positive results in dengue-endemic regions. Since 12% of the cohort was also dengue-positive and some patients had other co-infections, diagnostic overlap and difficulty in attributing complications solely to scrub typhus cannot be excluded. The absence of a control group and multivariable analysis also limited the assessment of independent predictors of complications and ICU admission.

Implications for practice and research**

Future multicenter studies with larger cohorts are required to better define the true burden of disease, explore regional variations, and identify predictors of severe outcomes. From a clinical perspective, the findings reinforce the importance of empirical doxycycline therapy in patients with acute febrile illness during the monsoon and post-monsoon seasons in endemic regions, even before confirmatory testing. Public health strategies must prioritize awareness campaigns, vector control measures, and affordable diagnostic tools to reduce the burden of scrub typhus in India. 

## Conclusions

Scrub typhus is an important cause of acute febrile illness in endemic regions and may present with diverse clinical manifestations, ranging from mild disease to severe multi-organ involvement. Fever was the most common presenting symptom in this study, while elevated liver enzymes, thrombocytopenia, and hyponatremia were frequent laboratory abnormalities. Respiratory complications such as acute respiratory distress syndrome and pneumonia were the most common severe manifestations, and a significant proportion of patients required intensive care support. Seasonal clustering during the monsoon and post-monsoon months highlights the importance of maintaining a high index of suspicion during this period. The favorable in-hospital outcomes observed in this cohort are consistent with the benefits of prompt recognition, doxycycline therapy, and supportive care. Further multicenter studies with larger sample sizes are required to better understand the disease spectrum and predictors of severe outcomes.
